# Altered patterns of displacement within the Achilles tendon following surgical repair

**DOI:** 10.1007/s00167-016-4394-5

**Published:** 2016-12-21

**Authors:** Åsa Fröberg, Ann-Sophie Cissé, Matilda Larsson, Mattias Mårtensson, Michael Peolsson, Tomas Movin, Anton Arndt

**Affiliations:** 1 Division of Orthopaedics and Biotechnology Karolinska University Hospital, Department of Clinical Sciences Intervention and Technology (Clintec), Karolinska University Hospital, Karolinska Institute, Huddinge, 141 86 Stockholm, Sweden; 2 School of Technology and Health, KTH Royal Institute of Technology, Huddinge, Sweden; 3 SICS, Swedish ICT Research Institute, Kista, Sweden; 4 Department of Clinical Science and Education, Södersjukhuset, Karolinska Institute, Stockholm, Sweden; 5 The Swedish School of Sports and Health Sciences, Stockholm, Sweden

**Keywords:** Achilles tendon, Deformation pattern, Rupture, Surgical repair, Speckle tracking

## Abstract

**Purpose:**

Ultrasound speckle tracking was used to compare tendon deformation patterns between uninjured and surgically repaired Achilles tendons at 14–27-month follow-up. The hypothesis was that the non-homogenous displacement pattern previously described in uninjured tendons, where displacement within deep layers of the tendons exceeds that of superficial layers, is altered following tendon rupture and subsequent surgical repair.

**Methods:**

In the first part of this study, an in-house-developed block-matching speckle tracking algorithm was evaluated for assessment of displacement on porcine flexor digitorum tendons. Displacement data from speckle tracking were compared to displacement data from manual tracking. In the second part of the study, eleven patients with previous unilateral surgically treated Achilles tendon rupture were investigated using ultrasound speckle tracking. The difference in superficial and deep tendon displacement was assessed. Displacement patterns in the surgically repaired and uninjured tendons were compared during passive motion (Thompson’s squeeze test) and during active ankle dorsiflexion.

**Results:**

The difference in peak displacement between superficial and deep layers was significantly (*p* < 0.01) larger in the uninjured tendons as compared to the surgically repaired tendons both during Thompson’s test (−0.7 ± 0.2 mm compared to −0.1 ± 0.1 mm) and active dorsiflexion (3.3 ± 1.1 mm compared to 0.3 ± 0.2 mm). The evaluation of the speckle tracking algorithm showed correlations of *r* ≥ 0.89 between displacement data acquired from speckle tracking and the reference displacement acquired from manual tracking. Speckle tracking systematically underestimated the magnitude of displacement with coefficients of variation of less than 11.7%.

**Conclusions:**

Uninjured Achilles tendons display a non-uniform displacement pattern thought to reflect gliding between fascicles. This pattern was altered after a mean duration of 19 ± 4 months following surgical repair of the tendon indicating that fascicle sliding is impaired. This may affect modulation of the action between different components of the triceps surae, which in turn may affect force transmission and tendon elasticity resulting in impaired function and risk of re-rupture.

## Introduction

Acute rupture of the Achilles tendon commonly occurs during sport activities and affects persons of working age and regardless of treatment choice there is a remaining decrease in performance in functional tests, range of motion, calf muscle circumference and in the physical activity level 12–24 months after injury [22, 23, 35].

It has been proposed that non-uniform stress across the tendon’s cross-sectional area may be an aetiological factor in Achilles tendon injury [2]. The Achilles tendon is formed by the aponeuroses of the medial and lateral gastrocnemius and soleus muscles and, although not in all individuals, the plantaris tendon. Fibres from the aponeuroses twist so that fibres from the lateral gastrocnemius end up on the ventral side of the tendon, medial gastrocnemius fibres become dorsal and soleus fibres become medial at insertion [3, 10, 11, 33]. Force in the Achilles tendon has been shown to be non-homogenous from medial to lateral during loading of different components of the triceps surae [2]. Ultrasound speckle tracking studies of healthy Achilles tendons have shown that displacement within ventral or deep parts of the tendons exceeds that of the dorsal or superficial parts during passive plantar- and dorsiflexion of the ankle [1, 29] and during treadmill walking [13]. Achilles tendons of middle-aged adults display more uniform displacement patterns as compared to young adults during ankle motion [30]. Ultrasound studies of surgically repaired Achilles tendons have shown increased thickness, decreased echogenicity [5, 21] and decreased gliding between the tendon and surrounding tissue [21, 25] which persisted for years after injury. In these studies, gliding and tendon motion was subjectively assessed. To our knowledge, ultrasound speckle tracking has not previously been used to study the deformation pattern in Achilles tendons that have been surgically repaired after tendon rupture.

Ultrasound speckle tracking offers a means to study deformation patterns within the tendon substance. It is a method to assess tissue motion based on tracking of unique patterns in ultrasound images created by interference of reflected ultrasound between a series of frames. Several speckle tracking algorithms have previously been validated for assessment of displacement in tendon tissue [8, 9, 19, 24, 36]. The majority of these algorithms have been custom-developed for application to tendon tissue because commercially available speckle tracking algorithms are not developed for this application.

The aim of this study was to use ultrasound speckle tracking to compare tendon deformation patterns between uninjured and surgically repaired Achilles tendons 14–27 months following suturing of the tendons. The hypothesis was that the non-homogenous displacement pattern normally seen in Achilles tendons is altered following surgically treated tendon rupture. In order to do this, an in-house-developed block-matching speckle tracking algorithm for analysis of displacement in tendon tissue was evaluated. Knowledge of how normal tendon biomechanics are affected by injury and healing may contribute to understanding the risk of re-rupture and residual decrease in tendon function following rupture.

## Materials and methods

In the first part of this study, an in-house-developed block-matching speckle tracking algorithm was evaluated for assessment of displacement on porcine flexor digitorum tendons. Fresh frozen porcine feet for the speckle tracking validation were purchased at the local food store. In the second part of the study, eleven patients with previous unilateral surgically treated Achilles tendon rupture were investigated using ultrasound based speckle tracking. Displacement in the surgically repaired and uninjured tendons was compared.

### Speckle tracking algorithm evaluation

**Fig. 1:**
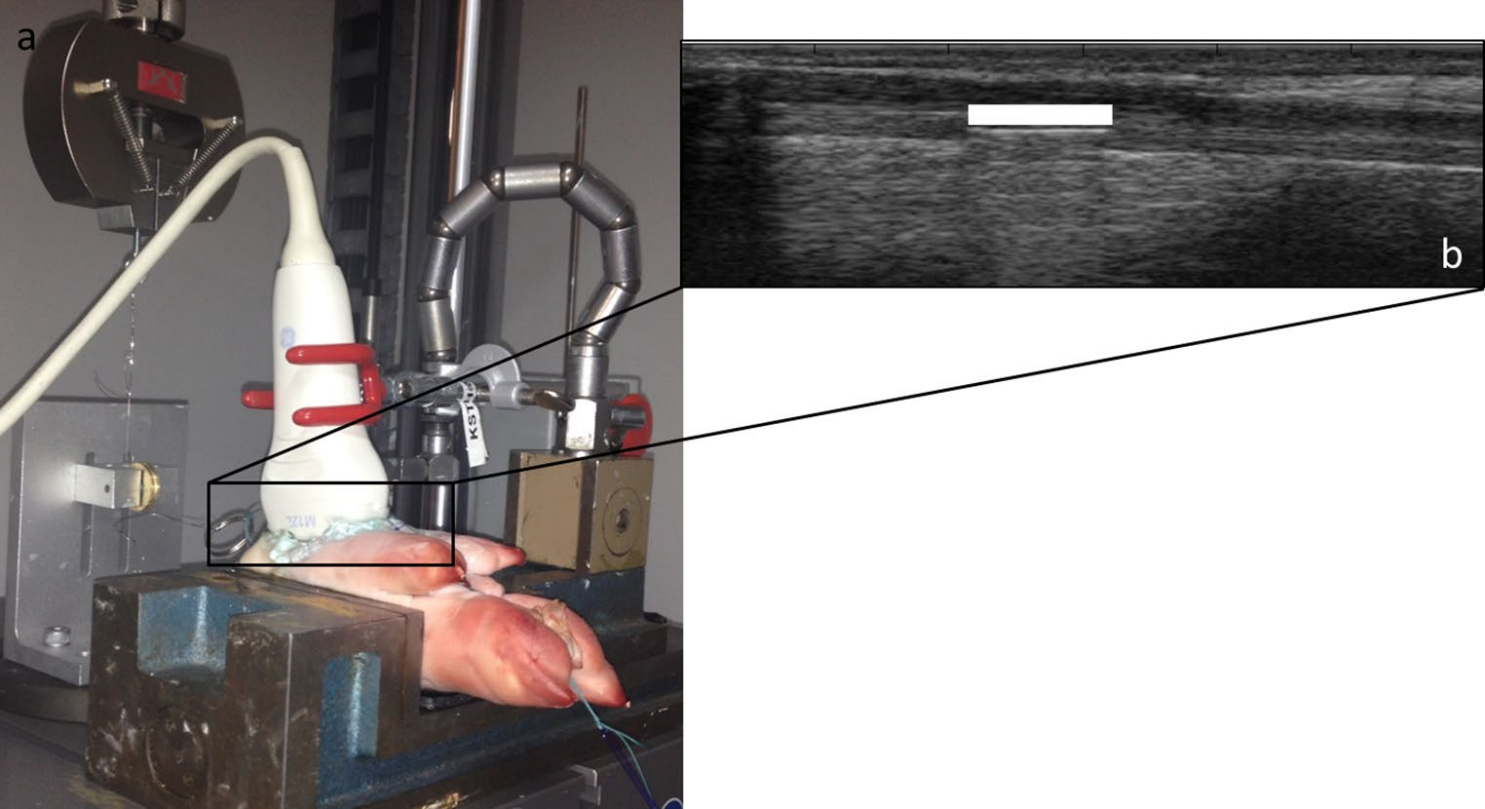
Experimental set-up speckle tracking algorithm evaluation. **a** Porcine foot mounted in a vice. The flexor digitorum tendon is attached to a materials testing machine via sutures and a stainless steel wire. An ultrasound probe is placed on the tendon. **b** Ultrasound image of the flexor digitorum tendon with inserted aluminium platelet and a superficial region of interest (ROI)

An in-house speckle tracking algorithm, previously developed for estimation of arterial wall strain [20], was modified to assess displacement in tendons. The algorithm was implemented in MATLAB (R2014a, MathWorks Inc., Natick, MA, USA) and applied on the envelope detected B-mode ultrasound data. 2D motion estimation was performed using a kernel size of 52*λ* (laterally) × 25*λ* (axially), 80% kernel overlap, and normalized cross correlation as similarity measure. The in-house-developed speckle tracking algorithm was evaluated on porcine flexor digitorum tendons using an experimental set-up similar to that described by Korstanje et al. [19]. A 5 × 5 mm aluminium platelet was surgically inserted into the flexor digitorum tendon of two porcine feet leaving the skin superficial to the tendon intact. Number 2 Ethibond^®^ sutures (Ethicon, Livingston, Scotland) were sutured to each tendon end. The proximal tendon suture was attached to a stainless steel wire, which in turn was attached to a materials testing machine (ElectroPuls E3000, Instron, Norwood, MA, USA) via a pulley. The distal tendon suture was attached to a 500 g weight (Fig. 1a). An M12L linear array transducer (GE Healthcare, Horten, Norway) connected to a Vivid 7 ultrasound machine (GE Healthcare) was covered with ultrasound gel and mounted over the flexor digitorum tendon with the inserted aluminium platelet (Fig. 1b). The materials testing machine was programmed to displace the tendon 5, 10 and 15 mm at 5, 10 and 15 mm/s, respectively. The displacement and speed of the materials testing machine were not used as reference due to mechanical losses in the set-up as has been reported elsewhere [19]. Five ultrasound acquisitions (14 MHz, 65.3 FPS, depth 4 cm) were made for each distance and speed. The test order was randomly assigned. The first and last frames of each motion sequence were manually identified, and files were converted to HDF format and imported into MATLAB (MathWorks Inc.). To find a reference displacement, both ends of the aluminium platelet were manually tracked frame by frame resulting in two displacement curves for each trial. Peak displacements of the two manual tracking displacement curves were identified, averaged and used as reference displacement for each trial. For speckle tracking assessment of displacement, a 5 mm region of interest (ROI) was placed superficial to the aluminium platelet (Fig. 1b) and displacement was analysed. Resulting displacement curves were saved in Excel (2013, Microsoft Corp., Redmond, WA, USA), and peak displacement was identified for each trial. Peak displacement from speckle tracking (d_st_) was compared to the corresponding reference displacement (d_ref_), and the mean and standard deviation (SD) of the absolute error (|d_ref_–d_st_|) in peak displacement were calculated for the five trials of tendons 1 and 2 at each displacement and speed. The coefficient of variation (SD/mean) was calculated for each condition for tendons 1 and 2, respectively. For each trial, peak displacement derived from speckle tracking was plotted against peak displacement from manual tracking using Origin (version 9.1, Microcal Inc., Northampton, MA, USA). A line was fitted to the plot, and the Pearson coefficient of correlation (*r*) was calculated.

### Post-operative follow-up

From February 2007 to April 2008, 68 patients with Achilles tendon rupture were treated at Karolinska University Hospital, Stockholm, Sweden. Patients aged <18 years or >65 years, or with re-ruptures, contralateral injuries, traumatic cuts, post-operative infection or who no longer lived in Stockholm were excluded. The remaining patients received information about the study by mail, and 10 males and one female (mean ± SD age: 50 ± 9 years, body height: 177 ± 10 cm, body mass: 82 ± 16 kg) agreed to participate. Post-operatively a lower leg plaster cast was applied for two weeks, followed by six weeks treatment with an ankle foot orthosis with an adjustable foot plate allowing successively increased range of motion. Weight bearing gradually increased after week two and was encouraged as tolerated after week six (Table 1). Patients participated in this study 19 ± 4 (14–27) months after surgery.

**Table 1:** Post-operative treatment protocol

Time	Treatment	Weight bearing
Day 1	Surgery	–
Week 1–2	Plaster cast in pf	Partial
Week 3–4	AFO locked at 30° pf, no ROM	Partial
Week 5–6	AFO with ROM between full pf and 10° pf	Partial
Week 7–8	AFO with ROM between full pf and 10° df	As tolerated
Week 9–14	Shoes with heel lifts of 1.5–2 cm	Full

*pf* plantarflexion, *df* dorsiflexion, *AFO* ankle foot orthosis with adjustable foot plate, *ROM* range of motion

Upon examination, patients filled out a general questionnaire and the Achilles tendon rupture score (ATRS). They then lay prone on an examination bed with both feet over the edge. The maximum active dorsiflexion of the ankle was manually measured using a goniometer bilaterally. An M12L linear array transducer (GE Healthcare) connected to a Vivid 7 ultrasound machine (GE Healthcare) was covered with gel and hand-held over the Achilles tendon. Bilateral longitudinal still images of the tendons were saved. The examiner then performed three repeated Thompson’s squeeze tests [28, 34] while an ultrasound acquisition was made on both the surgically repaired and uninjured tendon (14 MHz, 71.2 FPS, depth 3.5 cm). The patients then performed an active dorsiflexion starting at resting position to maximum dorsiflexion and then back to resting position. This was repeated three times for both legs.

Tendon thickness was measured from dorsal to ventral using the caliper function in EchoPAC 110.1.2 (GE Healthcare) on the longitudinal still images at the thickest point of the surgically repaired tendon and at the corresponding distance from the posterior process of the tibia in the uninjured tendon. For each trial, the first and last frames of each motion sequence were identified. Motion files were saved in HDF format and imported into MATLAB (MathWorks Inc.). Motion files were then blinded. Author ML assigned a random number to each file so that author ÅF performing the speckle tracking analysis did not know if the files came from a surgically repaired or uninjured tendon. A 20 mm ROI was placed in the superficial half of the tendon followed by a similar sized ROI in the deep half of the tendon. The thickness of the ROI was adapted between tendons to cover half the tendon thickness. Displacement curves for the deep and superficial parts of each tendon were plotted in Origin (Microcal Inc.), and peak displacements were manually identified and averaged over three trials. The difference in peak displacement between the deep and superficial parts was calculated for each tendon. The Stockholm Regional Ethics Committee approved the study (2008/547-31), and patients gave written informed consent.

### Statistical analysis

Mean and standard deviation of the difference in displacement were calculated for the surgically repaired and uninjured tendons, respectively. A two-sided paired *t* test was performed for the displacement in the superficial and deep parts of the surgically repaired and uninjured tendons during active dorsiflexion and Thompson’s test using MATLAB (MathWorks Inc.). The two-sided paired *t* test was repeated for the difference in displacement between the superficial and deep parts for the surgically repaired and uninjured tendon during active dorsiflexion and Thompson’s test. Mean displacement curves for superficial and deep displacement in the surgically repaired and uninjured tendon during active dorsiflexion and Thompson’s test were calculated using MATLAB (MathWorks Inc.). Tendon velocities were calculated as the derivative of the displacement curves for each tendon and condition using Excel (Microsoft Corp.).

## Results

### Speckle tracking algorithm evaluation

**Fig. 2:**
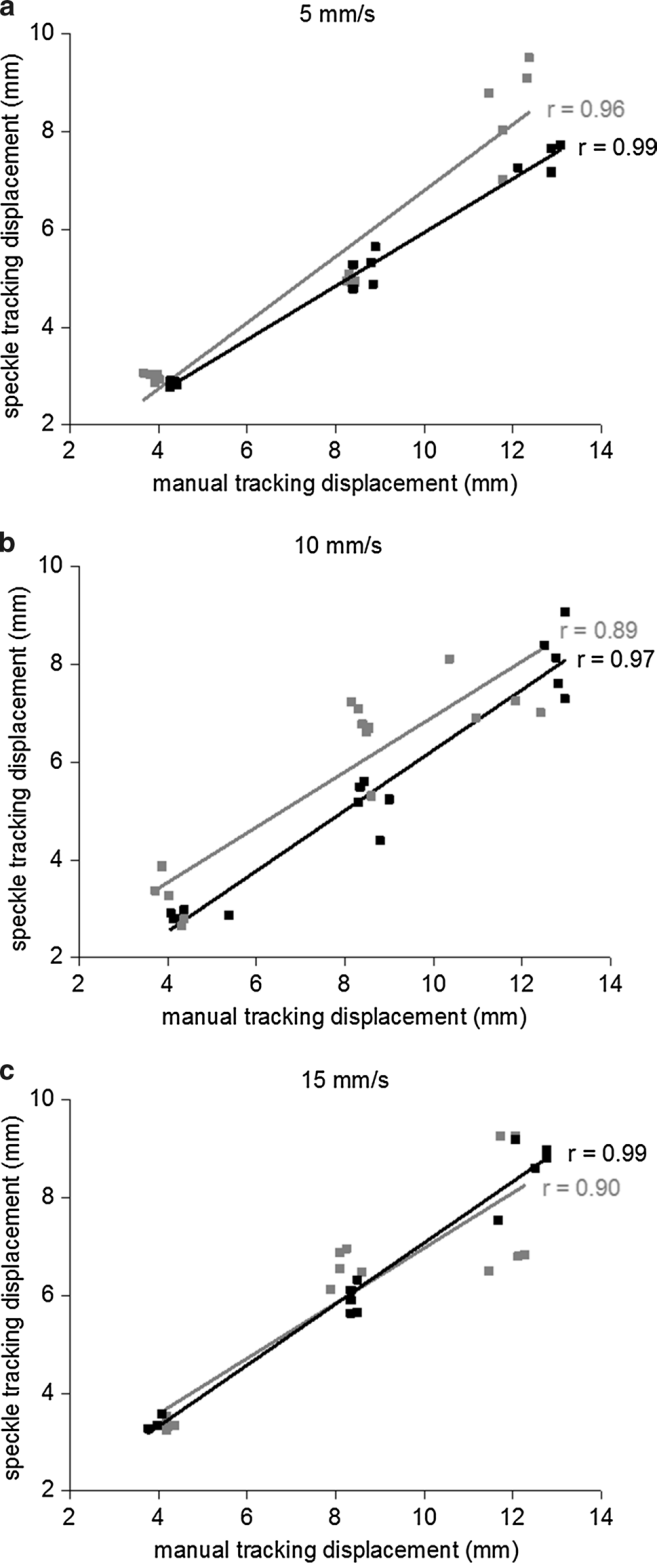
Correlations between speckle tracking displacement and reference displacement. Peak displacements (mm) as estimated by manual tracking and by speckle tracking at **a** 5 mm/s, **b** 10 mm/s and **c** 15 mm/s for porcine tendon 1 (*black*) and porcine tendon 2 (*grey*). Pearson coefficient of correlation (*r*) is given next to the graph. *p* < 0.01 for all correlations

There were significant (*p* < 0.01) linear correlations between speckle tracking displacement data and reference displacement for the three investigated velocities with *r* ≥ 0.89 (Fig. 2). The mean and standard deviation of the absolute errors in speckle tracking displacement data as compared to reference displacement for porcine tendons 1 and 2 are shown in Table 2, along with the coefficient of variation for speckle tracking displacement data. Due to difficulties in the manual tracking, three trials for porcine tendon 1 and one for porcine tendon 2 were removed. The removed trials belonged to different trial conditions, leaving at least four trials for the analysis of all displacements and velocities.

**Table 2:** Speckle tracking displacement error

	5 mm/s			10 mm/s			15 mm/s		
	Mean	CV_1_%	CV_2_%	Mean	CV_1_%	CV_2_%	Mean	CV_1_%	CV_2_%
5 mm	1.2 ± 0.4	2.1	2.7	1.1 ± 0.5	3.1	11.7	0.7 ± 0.2	3.8	3.3
10 mm	3.4 ± 0.2	6.8	1.4	2.4 ± 1.1	9.2	3.8	2.0 ± 0.6	5.0	5.0
15 mm	4.3 ± 1.1	3.7	11.5	4.4 ± 1.0	8.6	7.5	4.0 ± 1.0	7.5	18.1

Mean absolute error ± SD (mm) in speckle tracking displacement estimation for porcine tendons 1 and 2. The labels in the table correspond to the settings of the materials testing machine. The 5, 10 and 15 mm displacement settings resulted in 4.1, 8.4 and 12.1 mm displacements and the 5, 10 and 15 mm/s velocity settings resulted in 4.1, 7.7 and 11.5 mm/s as found by manual tracking. Speckle tracking underestimated displacement for all conditions. Coefficients of variation (%) are presented for porcine tendons 1 (CV_1_) and 2 (CV_2_) for each displacement and velocity

### Post-operative follow-up

**Fig. 3:**
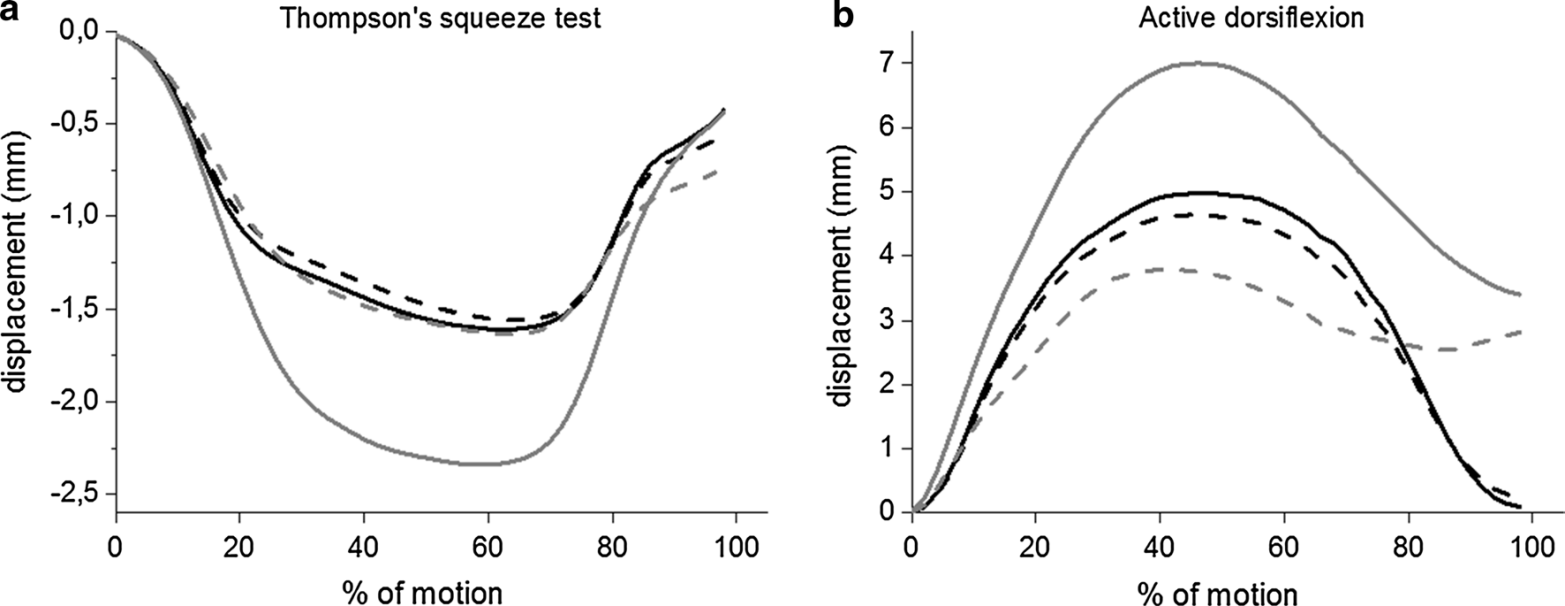
Mean Achilles tendon displacement. **a** Mean displacement in the superficial (*dashed*) and deep (*solid*) layers of the surgically repaired (*black*) and uninjured (*grey*) Achilles tendons during Thompson’s squeeze test for all patients. **b** Mean displacement in the superficial (*dashed*) and deep (*solid*) layer of the surgically repaired (*black*) and uninjured (*grey*) Achilles tendons during active dorsiflexion for all patients

Surgically repaired Achilles tendons had a mean ± SD dorsoventral thickness of 12.5 ± 2.0 mm and were significantly (*p* < 0.01) thicker than the uninjured tendons (5.6 ± 0.8 mm). The maximum active ankle dorsiflexion was 18° ± 7° and 18° ± 5° for the surgically repaired and uninjured sides, respectively. At follow-up, the median ATRS was 82 (29–99). One subject declined to answer the ATRS. Mean displacement curves for superficial and deep tendon layers for the surgically repaired and uninjured Achilles tendons during Thompson’s test and active dorsiflexion are shown in Fig. 3. The difference in peak displacement between superficial and deep layers was significantly (*p* < 0.01) larger in the uninjured tendons as compared to the surgically repaired tendons both during Thompson’s test and active dorsiflexion (Table 3). During Thompson’s test, displacement in the deep part of the tendons was significantly larger than in the superficial part (*p* < 0.01) for the uninjured tendons, whereas there was no significant difference for the surgically repaired tendons (Table 3). Displacement in the deep part of the tendons during active dorsiflexion was significantly larger than in the superficial part (*p* < 0.01) for both the uninjured and surgically repaired tendons (Table 3). During Thompson’s test, mean ± SD peak velocities were 10.0 ± 3.7 mm/s for the uninjured tendons and 9.2 ± 4.0 mm/s for the surgically repaired tendons, and during active dorsiflexion, mean ± SD peak velocities were 11.2 ± 2.8 mm/s for the uninjured tendons and 12.7 ± 1.8 mm/s for the surgically repaired tendons. Due to technical problems, the files for active dorsiflexion are missing for two subjects.

**Table 3:** Mean Achilles tendon displacement

	Superficial disp (mm)	Deep disp (mm)	*p* disp sup versus deep	Δ disp (mm)	*p* Δ disp SR versus uninj
**Thompson’s squeeze test**					
Surgically repaired					*p* < 0.01
Mean ± SD	−1.6 ± 0.8	−1.7 ± 0.8	n.s.	−0.1 ± 0.1	
Uninjured					
Mean ± SD	−1.7 ± 0.6	−2.4 ± 0.6	*p* < 0.01	−0.7 ± 0.2	
Active dorsiflexion*					
Surgically repaired					*p* < 0.01
Mean ± SD	5.0 ± 2.7	5.3 ± 2.8	*p* < 0.01	0.3 ± 0.2	
Uninjured					
Mean ± SD	4.0 ± 4.4	7.2 ± 4.1	*p* < 0.01	3.3 ± 1.1	

Mean displacement (mm) in the superficial and deep layers of the surgically repaired and uninjured tendon of all patients during the Thompson’s squeeze test and active dorsiflexion. Δ disp = difference in displacement between the deep and superficial layer

*SR* surgically repaired

* *n* = 9

## Discussion

The most important finding of the present study was that previously ruptured and surgically repaired Achilles tendons have a more uniform displacement pattern than uninjured tendons, up to 27 months after surgery. In the uninjured tendons, a non-uniform displacement pattern was observed, as has been described previously [1, 13, 29, 30]. The dorsoventral thickness of the injured tendons was significantly larger than that of the uninjured tendons, which is in agreement with previous studies [5, 21].

The evaluation of the speckle tracking algorithm showed that there was a strong correlation between displacement data acquired from speckle tracking and the reference displacement acquired from manual tracking (*r* ≥ 0.89). Speckle tracking systematically underestimated the magnitude of displacement (Table 2; Fig. 2). Mean peak displacements in the Achilles tendon ranged between 1.6 ± 0.8 and 7.2 ± 4.1 mm (Table 3), and mean peak velocity ranged between 9.2 ± 4.0 and 12.7 ± 1.8 mm/s for the different conditions. Within these ranges of displacement and velocity, the coefficient of variation was less than 11.7% for both porcine tendons. It therefore appears as if the systematic underestimation of displacement is relatively constant. A tendency for underestimation of displacement by ultrasound speckle tracking on B-mode images has previously been reported [8, 9, 36]. A likely explanation for this is out of plane motion causing tracking to fail momentarily, which will lead to underestimation of displacement. Since displacement estimation was made perpendicular to the ultrasound beam, it was dependent on the lateral resolution of the image which makes tracking more difficult. Validation was performed on porcine flexor digitorum tendons which are thinner than human Achilles tendons. As the Achilles tendons were thicker than the porcine tendons, larger ROIs could be used which presumably improved tracking as more speckles were included. Larger ROIs have previously been shown to improve tracking quality [15]. The intra- and inter-observer reliability were previously investigated by our research group for another block-matching speckle tracking algorithm, and an average intraclass correlation coefficient of 0.94 was reported [1]. In the study population of 11 patients, displacement in superficial and deep parts of the uninjured tendons could be distinguished with statistical significance which indicates that the method is clinically applicable.

Tendon fascicles are separated by loose connective tissue which enables the fascicles to slide past each other [4]. Lubricin is a mucinous glycoprotein which facilitates tendon gliding, and the expression of Lubricin in tissue is stimulated by shear forces [32]. Lubricin has been shown to be present at the interfaces of human Achilles tendon fascicles with higher concentration in the distal-free tendon than in more proximal parts, indicating that sliding between fascicles is normally more pronounced distally [32]. The non-uniform displacement demonstrated by ultrasound speckle tracking in the distal-free part of uninjured Achilles tendons has been suggested to reflect sliding between the separate fascicles originating from the muscles of the triceps surae [1, 29]. Normal tendon morphology is altered by tendon rupture, open repair and scar tissue formation, and the more uniform displacement pattern seen in the surgically repaired tendons in this study is presumably explained by a decreased ability of tendon fascicles to slide relative to each other.

In the supraspinatus tendon, it has been shown that structurally independent fascicles slide relative to each other to maintain tension throughout the tendon at varying joint angles [12]. During isometric plantarflexion of the ankle, displacement of the aponeurosis of the medial gastrocnemius exceeds displacement of the soleus aponeurosis when the knee is straight, whereas the opposite is true when the knee is flexed to 125° [6]. This difference in displacement indicates that there is considerable shear potential during plantarflexion which differs in direction depending on knee joint angle [6]. In vitro loading of different components of the triceps surae leads to non-uniform forces in the Achilles tendon, where loading of the medial gastrocnemius results in significantly higher forces in medial portions of the tendon and loading of the entire triceps surae leads to significantly higher forces in lateral portions [2]. Franz et al. [14] demonstrated that differential displacement between superficial and deep tendon layers is correlated with peak triceps surae moment, work and power during push-off in walking. In surgically repaired tendons, fascicle sliding seems to be impaired and this may have a negative effect on the modulation of the action of the different components of the triceps surae and on the optimization of force transmission at different knee and ankle joint angles. Furthermore, altered displacement patterns in tendon tissue will modify tendon function in stretch–shortening exercise and can be assumed to decrease tendon elasticity, both of which are factors which may predispose the tendon to future rupture.

The altered displacement pattern observed in the surgically repaired tendons in this study was present at a follow-up of 19 ± 4 months after rupture. Previous ultrasound studies of surgically repaired Achilles tendons have shown decreased echogenicity, disturbed striated appearance in the scar tissue and decreased gliding between the tendon and surrounding tissue persistent at follow-up times of up to 11 years after surgery [5, 21, 25]. In a study of Achilles tendon ruptures in rats, it was demonstrated that allowing the rats to run in a wheel without any form of immobilization resulted in more mature repair tissue with organized parallel collagen fibres and less presence of inflammatory cells as compared to rats immobilized with a plaster cast [7]. In patients with Achilles tendon rupture, it has been suggested that the use of semi-mobile orthoses lowers the risk of re-rupture as compared to rigid cast fixation in both surgically and non-surgically treated patients [18, 31]. Exercise applying tension to healing Achilles tendons has been shown to result in a higher tendon elastic modulus at five weeks after rupture, which in turn is correlated with better muscle function at 18 months [26, 27]. Little is known of the effect of tendon motion and loading on repair tissue and morphology in human Achilles tendons. It can be speculated that repair tissue with organized parallel collagen fibres may improve gliding properties and that this may contribute to better tendon function. A topic of future study is how deformation patterns in the Achilles tendon are affected following non-surgical treatment. There is a possibility that the surgical trauma itself affects gliding properties.

There are some limitations to the study. The median ATRS score was 82 with a range of 29–99 after a mean duration of 19 ± 4 months which is somewhat lower than previously reported [22, 27]. This may indicate a selection bias, and it can be speculated that patients who were less satisfied with their recovery were overrepresented in the study. Patients in this study had a median age of 50 years, which is higher than reported in previous studies of Achilles tendon rupture where the median age ranges between 42 and 47 years [16, 17]. Displacement patterns in the Achilles tendon have been shown to become more uniform with age [30], so the relatively high age in this study population may have affected the results of the uninjured tendons and differences between uninjured and surgically repaired tendons may be even larger in younger patients. The active dorsiflexion was not standardized other than that patients were instructed to perform a maximum dorsiflexion. However, there was no difference in the mean range of ankle motion between the uninjured and surgically repaired sides.

In surgically repaired tendons, fascicle sliding was impaired up to 27 months after surgical repair of the tendon, which may contribute to the known impaired function during activity long after surgery. It may be necessary to introduce new rehabilitation protocols into clinical practice to reinstate functional non-uniform displacement in the Achilles tendon post-operatively.

## Conclusions

Uninjured Achilles tendons display a non-uniform displacement pattern thought to reflect gliding between fascicles. This pattern was disturbed after a mean duration of 19 ± 4 months after surgical repair of the tendon.
